# Mechanochemical mineralization of “very persistent” fluorocarbon surfactants ‒ 6:2 fluorotelomer sulfonate (6:2FTS) as an example

**DOI:** 10.1038/s41598-017-17515-7

**Published:** 2017-12-07

**Authors:** Mengnan Lu, Giovanni Cagnetta, Kunlun Zhang, Jun Huang, Gang Yu

**Affiliations:** 1 State Key Joint Laboratory of Environment Simulation and Pollution Control (SKJLESPC), Beijing Key Laboratory of Emerging Organic Contaminants Control (BKLEOCC), School of Environment, POPs Research Center, Tsinghua University, Beijing, 100084 P. R. China; 2 Jinhua Polytechnic, Jinhua, 321007 China

## Abstract

Fluorinated organic chemicals have a wide variety of industrial and consumer applications. For long time perfluorooctane sulfonate and perfluorooctanoic acid have been used as precursors for manufacture of such chemicals. However, these C_8_ chain compounds have been demonstrated to be toxic, persistent, and bioaccumulative, thus inducing their phase-out. Currently, C_6_ telomer based fluorocarbon surfactants are considered better alternatives to C_8_ products because of their low bioaccumulability. But, their high persistency suggests that in the near future their concentrations will increase in the environment and in industrial waste. Being a solid state non-thermal technology, mechanochemical treatment is a good candidate for the destruction of emerging C_6_ fluorotelomers in solid waste. In the present study, 6:2 fluorotelomer sulfonate is effectively destroyed (~100%) in rapid manner (<1 h) by high energy ball milling with KOH. Stoichiometric fluoride formation confirms its entire mineralization, assuring that no toxic by-products are generated. Reaction mechanism and kinetics indicate that effective mineralization of the perfluorinated moiety is obtained thanks to a rapid CF_2_ “flake-off” process through radical mechanism.

## Introduction

**Figure 1:**
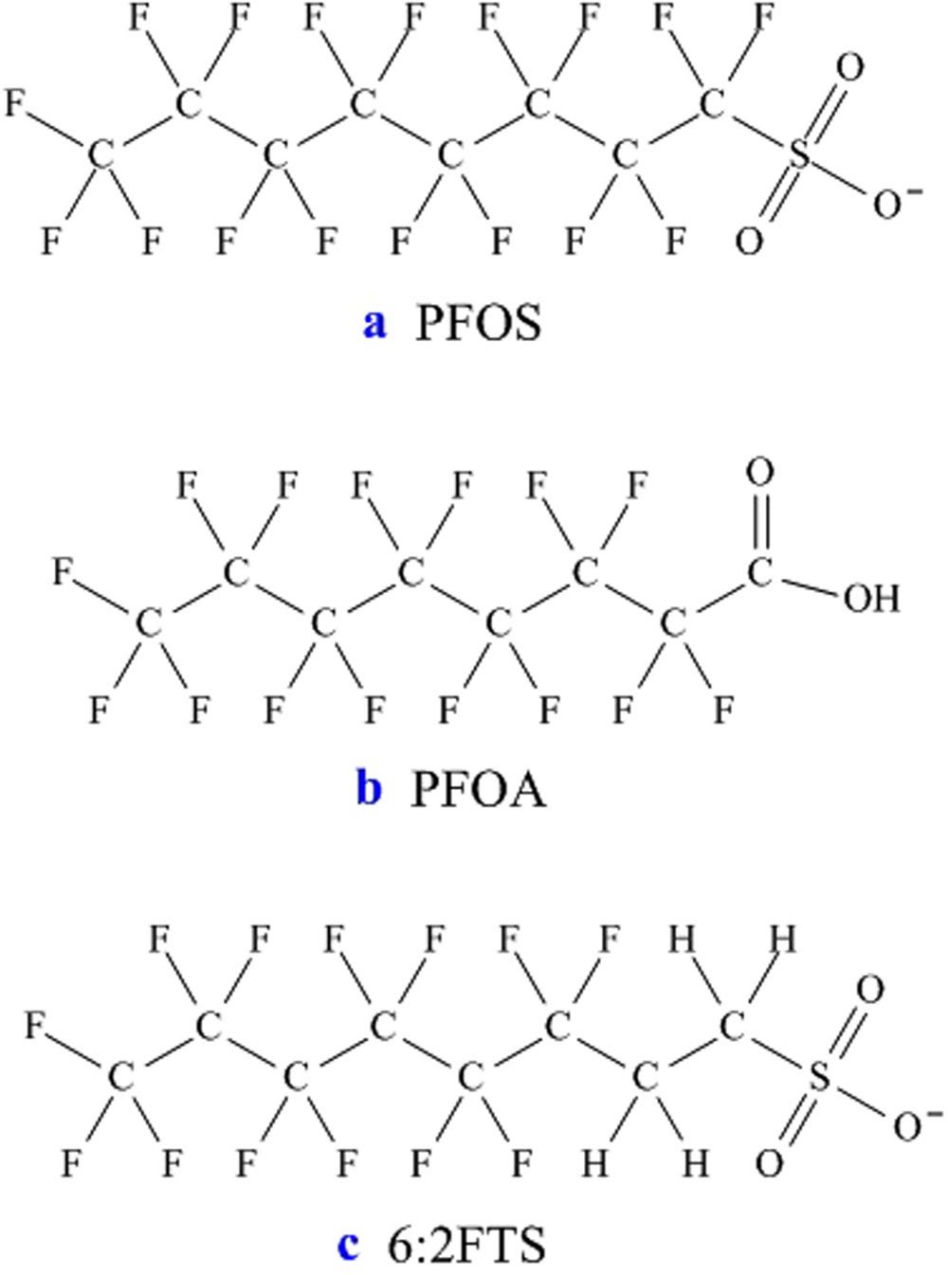
Chemical structure of (**a**) perfluorooctane sulfonate, (**b**) perfluorooctanoic acid, and (**c**) 6:2 fluorotelomer sulfonate.

Perfluoroalkyl substances (PFASs) are chemicals constituted by a perfluorinated carbon chain with a polar group. The stable C‒F bonds^1^ donate to these molecules peculiar properties such as hydro- and oleophobicity, tensioactivity, strong acidity, as well as a remarkable chemical stability. For this reason, they are utilized for the production of a large number of industrial and consumer products, like firefighting foams, perfluorinated polymers, pesticides, or liquid-repellent fabrics. However, many PFASs are being found to pollute many environmental compartments as a consequence of, e.g., uncontrolled release from manufacturing plants^2,3^, leaching from waste materials^4,5^, or use of fluorine-based firefighting products^6^. In addition, more and more PFAS molecules are being recognized to be persistent, bioaccumulative, and toxic^7,8^. In particular, perfluorooctane sulfonate (PFOS, Fig. 1a) and perfluorooctanoic acid (PFOA, Fig. 1b) have been massively utilized as precursors for manufacture of the above-mentioned products. Today, they are ascertained to be harmful to humans and the environment^9^. PFOS was listed as a persistent organic pollutant (POP) in 2009 by the Stockholm Convention^10^; PFOA was very recently (October 2017) recommended for inclusion in the POPs list^11^. Therefore, long chain PFASs are being phased out in many countries.

Unfortunately, currently there are no valid non-fluorine-based substitutes for PFASs, so manufacturers are shifting their production toward shorter chain ones (with six or less perfluorinated carbon atoms). These homologues are believed to be less bioaccumulative and, so, with less negative effects on human health. Nonetheless, they can be as persistent and toxic as the long chain surfactants^12^. In particular, persistence is the most important single factor, because PFAS extreme stability determines poorly reversible contamination in the environment (especially in water) that leads to continuous exposure to such chemicals, similarly to bioaccumulative compounds^13^. For this reason, concerns on “very persistent” (vP) chemicals have been raised, both at scientific and regulatory level^14,15^.

C_6_ telomer-based fluorosurfactants are becoming predominant alternatives for various applications that formerly required the use of PFOS or PFOA^16^. For example, 6:2 fluorotelomer sulfonate (6:2FTS, Fig. 1c) is being employed as a polymer processing aid in the synthesis of fluoropolymers^17^; it is also widely utilized as chrome mist suppressants in electroplating industry^18,19^, or as co-formulant in aqueous film-forming foams^20^. Currently, available data suggest that 6:2FTS is neither highly toxic nor bioaccumulative in aquatic ecosystems, according to regulatory criteria^12,21^. However, as its biodegradation is extremely slow and incomplete^22^, it can be considered a vP substance^14^. In view of its potential risk, several studies have reported the degradation of 6:2FTS in aqueous phase. For example, Yang *et al*.^19^ investigated its treatment by typical advanced oxidation processes, demonstrating that 6:2FTS can be mineralized in rapid manner by UV irradiation combined with H_2_O_2_. Likewise, Zhuo *et al*.^23^ achieved 6:2FTS electrochemical oxidation on a Ti/SnO_2_-Sb_2_O_5_-Bi_2_O_3_ anode, while PFOS was recalcitrant to such treatment. In both studies some intermediates (i.e. shorter-chain perfluorocarboxylic acids) were identified, which are known to be refractory to the attack of •OH radicals generated by these treatments^24^. In sum, both works clearly prove that, in spite of their vP, fluorotelomers can be treated effectively and therefore their release into the environment can be controlled in feasible manner. In this sense, fluorotelomers are more environmentally friendly than the C_8_ PFASs (in particular, PFOS).

To the best of our knowledge, there is no report on the destruction or decomposition of fluorotelomer alternatives in solid waste (e.g. sludge from chrome-plating industry, spent adsorbents from wastewater clean-up). Nevertheless, disposal of PFAS-containing waste is being regulated in stricter manner by governments^25^, thus causing additional high costs. A cheap non-combustion solid state technology is highly required to ensure PFAS safe destruction in industrial waste and prevent secondary contamination by leaching and percolation.

In the last two decades, mechanochemical (MC) methods have been widely recognized as powerful tools for environmental applications such as special material preparation and waste treatment^26,27^. Especially, MC destruction is a promising non-combustion technology for the disposal of halogenated POPs^28^. It is realized at environmental temperature by high energy milling of the organic pollutants with a co-milling reagent that boosts the reaction rate. Thanks to its solvent-freeness, simple plant design, and easiness of reaction control, MC destruction is considered a green technology^29,30^. A number of chlorinated and brominated organic pollutants were efficaciously mineralized into halides and amorphous carbon by high energy milling. Mineralization is a key requirement for halogenated organic pollutant destruction technologies, in order to prevent release of unintentionally generated POPs, like dioxins. Newly published works demonstrates that dioxins can be formed during MC treatment of halogenated organics due to mechanothermal effect, but they are eventually destroyed after sufficiently long milling time^31–33^.

In their early work, Shintani *et al*.^34^ confirmed the MC destruction of PFOS and PFOA by co-milling with CaO under very energy intensive conditions (i.e. 700 rpm main disk rotation speed), but they could not prove in a straightforward way the complete mineralization of both perfluorinated compounds. To this aim, we used in two previous works potassium hydroxide as reagent under mild intensity conditions (i.e. 275 rpm rotation speed). Stoichiometric fluoride recovery demonstrated the effectiveness of MC treatment with KOH to destroy PFOS, PFOA, and other perfluorinated compounds^35,36^. Yet, the really elevated pH of the milled residue suggested us to find alternative ways. Later, we succeed to implement a Waste-to-Materials approach to destroy several perfluorinated chemicals to generate a useful fluorinated inorganic compound of industrial interest (i.e. LaOF) by co-milling with stoichiometric amount of La_2_O_3_^37^. The developed MC reaction can be realized with almost pure PFASs to obtain a ~100% conversion product of high added-value, which justifies the employment of the expensive La_2_O_3_ as reagent. Obviously, such approach is not suitable for treatment of PFAS-contaminated waste. Cheap and expandable hydroxides are actually suitable reagents for waste disposal by MC treatment. Besides, it was verified that lower amounts of hydroxides are effective in combination with other reagents, like persulfate^38^, thus pH of the final residue is contained.

The present study is aimed to achieve the MC mineralization of C_6_ telomer fluorocarbon surfactants with KOH, using 6:2FTS as model compound. In particular, attention is paid to the presence in the molecule of a hydrogenated moiety, which determined an unusual degradation pathway that is different from those previously investigated. This work is the first step to assess the feasibility of MC destruction as potential treatment technology for industrial solid waste containing vP fluorotelomers, whose importance in industry and presence in the environment are increasing.

## Results and Discussion

### FTS mechanochemical destruction

**Figure 2:**
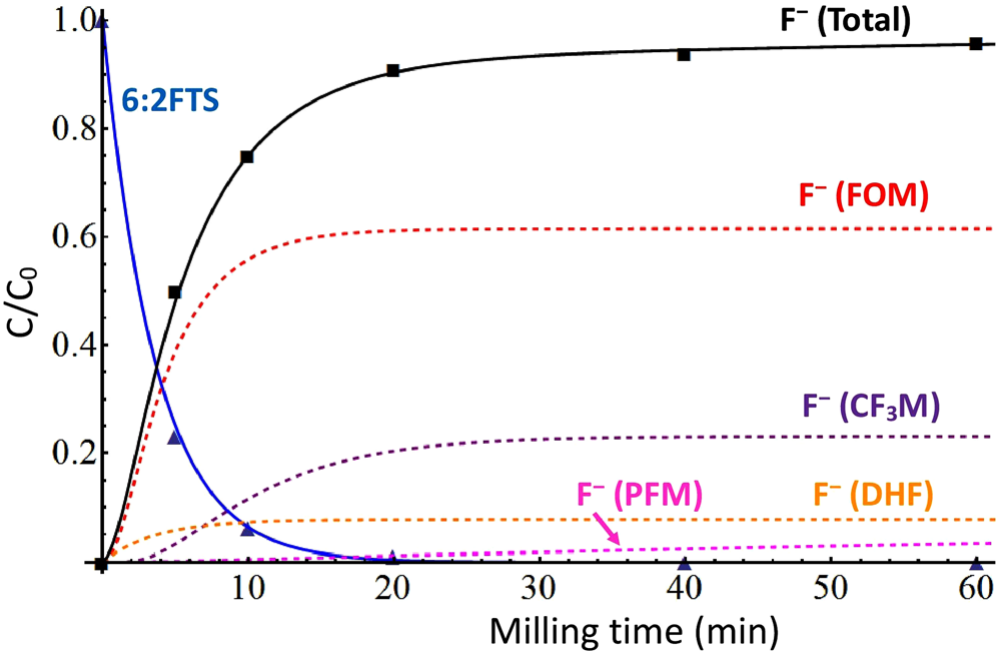
Kinetics of 6:2FTS MC destruction and fluoride build-up (with its components estimated by the reaction model).

6:2FTS was co-milled with KOH in a planetary ball mill. Experimental results corroborate the fast degradation of 6:2FTS (Fig. 2). After 20 min milling, the fluorinated compound is almost entirely depleted. In our previous work on PFOS MC destruction^36^, where milling and reaction conditions were the same employed in the present study, we found a much slower degradation rate. This strongly suggests that the presence of hydrogens in the alkyl chain introduces a weak point in the molecule. Stoichiometric recovery of fluorides from the reaction products confirms the effective mineralization of 6:2FTS. This is a key achievement to ensure that no halogenated organic by-products are generated during the reaction. PFAS destruction technologies can generate two kinds of by-products that are known to be harmful, i.e. volatile light perfluorinated compounds and fluorinated dioxins^39,40^. For instance, incineration of PFOS or PFOA has to be conducted with adequate amount of CaO to avert release of light fluorinated compounds^41^. Inorganic fluoride formation implies that no toxic by-products are released during ball milling. Contrary to expectations, ion chromatographic analysis showed that sulphate recovery was only 10% of the theoretical amount, while a stoichiometric yield was obtained after the PFOS MC destruction^36^. This result indicates two peculiar facts that are different from previous findings (as discussed later): 6:2FTS MC destruction mainly does not begin with sulfonate group detachment, and very likely the hydrogenated moiety is more resilient to MC destruction.

**Figure 3:**
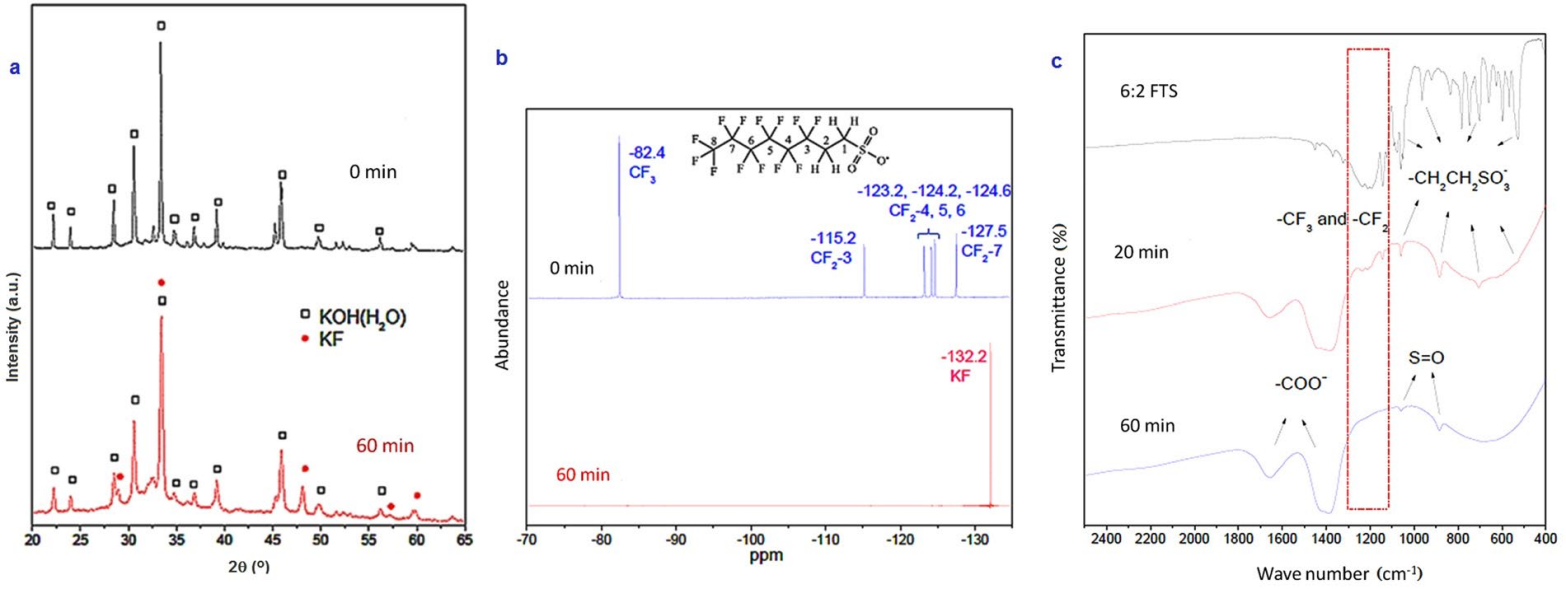
(**a**) XRD pattern, (**b**) ^19^F NMR spectra, and (**c**) FTIR spectra of 6:2FTS MC destruction at different milling time.

Chief crystalline phases involved in the MC reaction were identified by XRD diffraction (Fig. 3a). Diffractogram of the reagents shows solely the pattern of KOH (JCPDS card No. 15-0890) because 6:2FTS quantity in the reaction mixture is relatively low. After 60 min milling, KOH and KF∙2H_2_O (JCPDS card No. 01-0854) diffraction peaks are detected in the powder product, thus further confirming that mineralization of the fluorinated compound was achieved. In order to confirm the complete transformation of organic fluorine (which is not detected by XRD) into inorganic one, NMR analysis was executed as well.

NMR is an efficacious technique to identify structure of fluorinated compounds^42^. ^19^F has a nuclear spin of 1/2 and an elevated magnetogyric ratio, which means that this isotope is highly responsive to NMR measurements. Furthermore, ^19^F comprises 100% of naturally-occurring fluorine^43^. ^19^F NMR spectra of the unmilled sample shows six organic fluorine peaks that correspond to specific positions in the 6:2FTS molecule (Fig. 3b). Specifically, the peak at −82.4 ppm is assigned to CF_3_ group^44,45^, with a response area 1.5 times larger than those of other fluorinated groups owing to CF_3_ three fluorine atoms^46^. The resonance peak at −115.2 ppm is ascribed to the CF_2_ at position 3, bounded with CH_2_. The last four resonance peaks at −123.2, −124.2, −124.6, and −127.5 ppm correspond to intermediate positions of the alkyl chain (i.e. C_4_, C_5_, C_6_, and C_7_, respectively). The response of organic fluorine totally disappeared in ^19^F NMR spectrum of the sample ball milled for 60 min. Conversely, a very strong peak at −132.2 ppm attributed to inorganic fluoride appears, due to KF. In sum, NMR outcome confirms the rapid and entire mineralization of 6:2FTS in 1 h high energy ball milling.

Additional analyses were conducted to identify intermediates and products of 6:2FTS MC destruction. Molecular transformations of the telomeric compound were identified by FTIR (Fig. 3c). Perfluorinated chain response is in the range of 1150–1300 cm^−1^, ascribed to stretching of C‒F bonds in CF_3_ and CF_2_^47^. Vibrations of the sulfonic and methylenic moieties are in the 500–1100 cm^−1^ range. The intensity of such characteristic peaks decreases in 20 min-milled sample, and eventually disappear after 60 min milling. At the same time, two bands appear at 1400 cm^−1^ and 1680 cm^−1^, which are attributed to C‒O vibrations and ‒COO^−^ asymmetrical vibration, respectively^48^. In accordance with previous findings^35,36^, such peaks are mainly due to potassium formate. Furthermore, S=O vibration signals (880 and 1040 cm^−1^) are clearly identifiable in 60 min-milled samples, which might be due to generation of sulphate. FTIR results therefore confirm mineralization of the 6:2FTS perfluorinated moiety and suggest that carbon atoms are transformed into HCOO^−^ as sole product.

**Figure 4:**
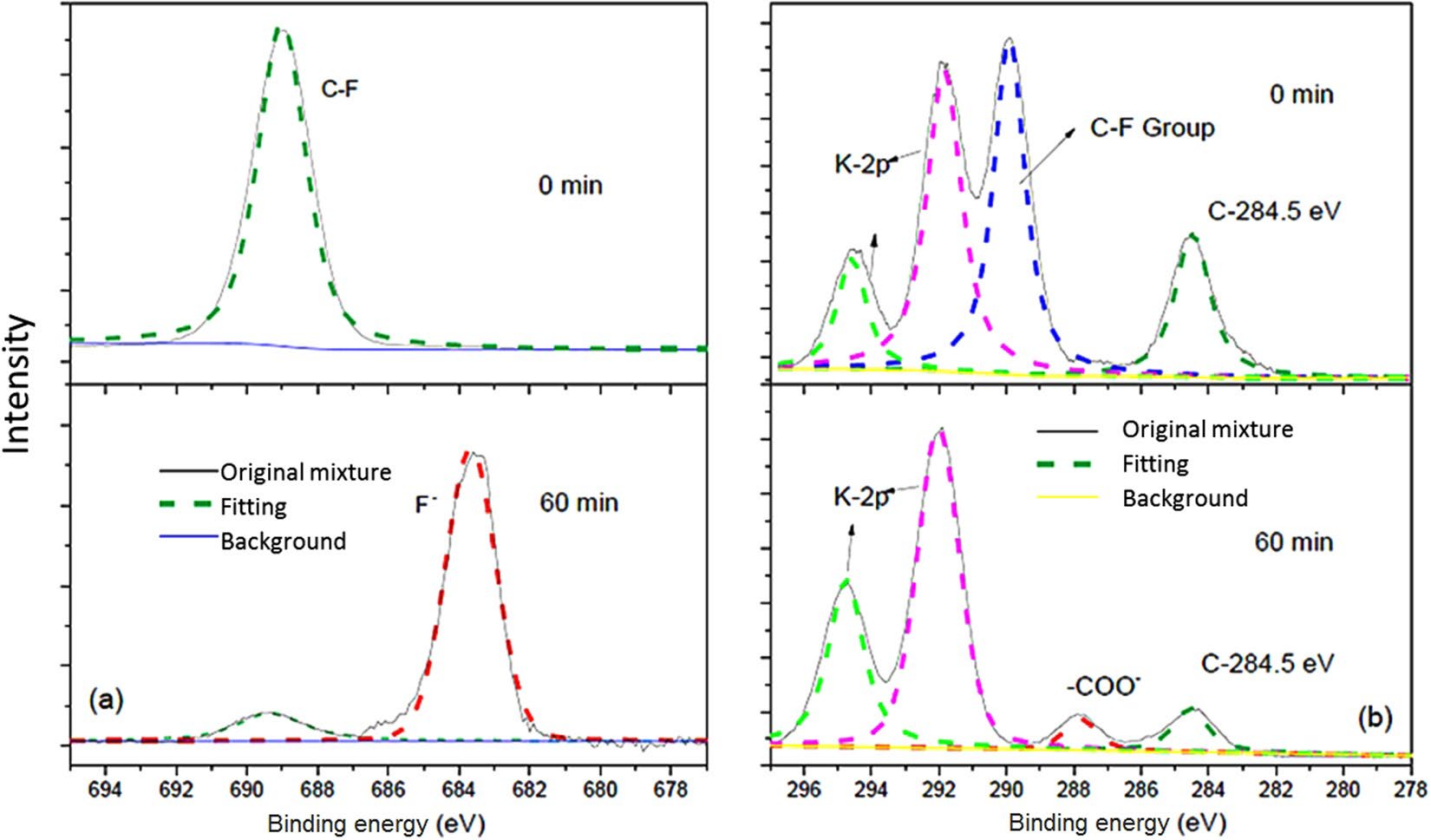
(**a**) F1s and (**b**) C1s XPS spectra of unmilled and 60 min-milled samples.

In order to corroborate this latter finding, XPS analysis was also performed on unmilled and 60 min-milled samples (Fig. 4). The peak at 689.0 eV in the F1s region is ascribed to organic fluorine^49^. The notable reduction of its intensity after 60 min milling, together with the appearance of F^−^ signal (684.0 eV), substantiates once more the occurred mineralization of the perfluorinated chain. C1s XPS spectrogram shows the presence of typical C‒F peak (290.0 eV), another signal that can be attributed to C‒C chain structure and occurrence of adventitious carbon (284.5 eV), and two peaks that derive from the massive presence of potassium (292.0 eV and 295.0 eV). Consistently with F1s spectrograms, the C‒F signal fades in the 60 min-milled sample, while both K2p peaks remain unchanged. Likewise, the C‒C chain structure signal intensity is lowered, suggesting that only interference from adventitious carbon is detected. It should be noted here that such residual signal can be only attributed to adventitious carbon: although amorphous carbon is a common product of MC destruction of (fluorinated) organic molecules^28,37^, neither experimental evidences (e.g. the colour of milled material was not dark) nor previous works^35,36^ prove the existence of carbonaceous matter among the products. Coherently, a signal corresponding to carboxylate comes out in the milled sample that very likely indicates presence of formate, in agreement with FTIR outcome.

**Figure 5:**
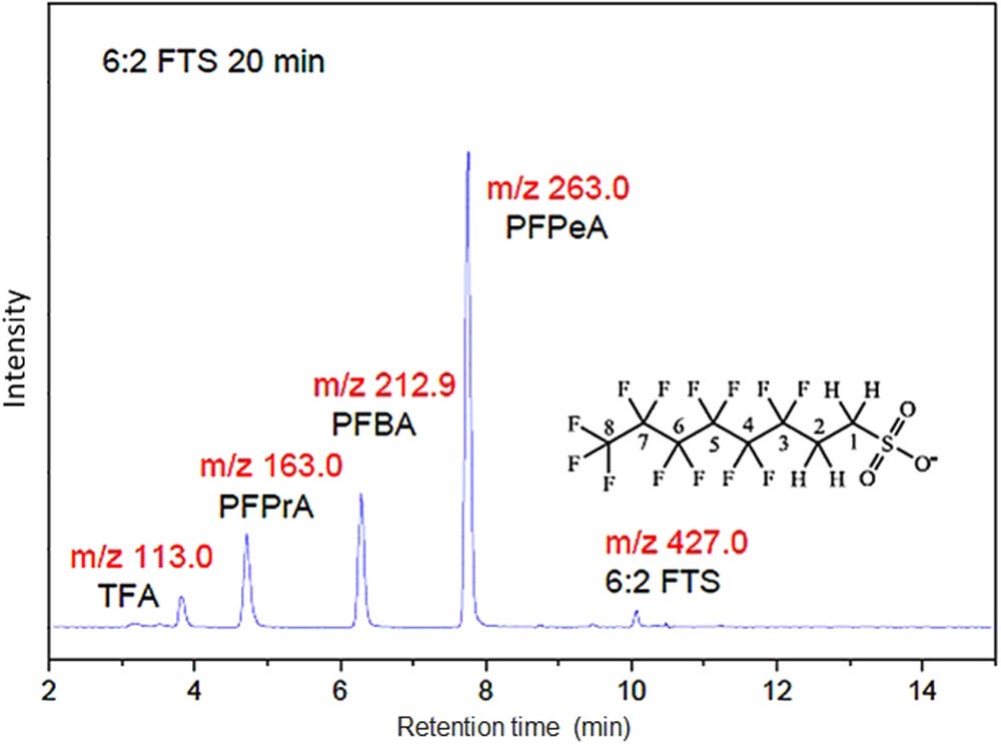
Intermediates of 20 min-milled 6:2FTS with KOH identified by LC-MS-MS.

MC reaction intermediates were identified by LC-MS-MS (Fig. 5). At 20 min milling, four intermediates were identified, viz. m/z = 263.0, 212.9,163.0, and 113.0 that are assigned to perfluoropentanoic acid (PFPeA), perfluorobutanoic acid (PFBA), perfluoropropionic acid (PFPrA) and trifluoroethanoic acid (commonly called trifluoroacetic acid, TFA), respectively. Their presence strongly corroborates the “flake-off” of CF_2_ moieties as main degradation mechanism of the perfluoroalkyl chain.

### Reaction mechanism

MC destruction of organic compounds usually begins with the MC activation of the co-milling reagent (an inorganic compound). The enhanced reactivity of reagents is a direct consequence of the physicochemical transformations that are provoked by intensive mechanical action, i.e. particle crushing, heating, build-up of crystalline defects, amorphization, etc. For example, high energy milling induces generation of trapped electron on metal oxide particle surfaces due to oxygen vacancy accumulation^50^; silica (SiO_2_) crystal crushing produces new electron-rich surfaces because of homolytic rupture of Si‒O bonds^51,52^; zero valent metals can effectively react with organics thanks to the non-oxidized fresh surfaces formation and rapid mixing caused by ball milling^53,54^. All these reagents have been utilized successfully to destroy halogenated organic pollutants because they can transfer electrons to organic pollutants^28^. Hydroxides are different: they react directly with the organic pollutant, taking advantage of the force field acting on the organic molecules that increases their reactivity. Specifically, mechanical forces determine a reduction of the frontier orbital energy gap^55^ and also decrease the activation energy of chemical reactions^56^. Thus, MC destruction with hydroxide mainly occurs because of MC activation of the organic pollutants, which then react with OH^−^.

**Figure 6:**
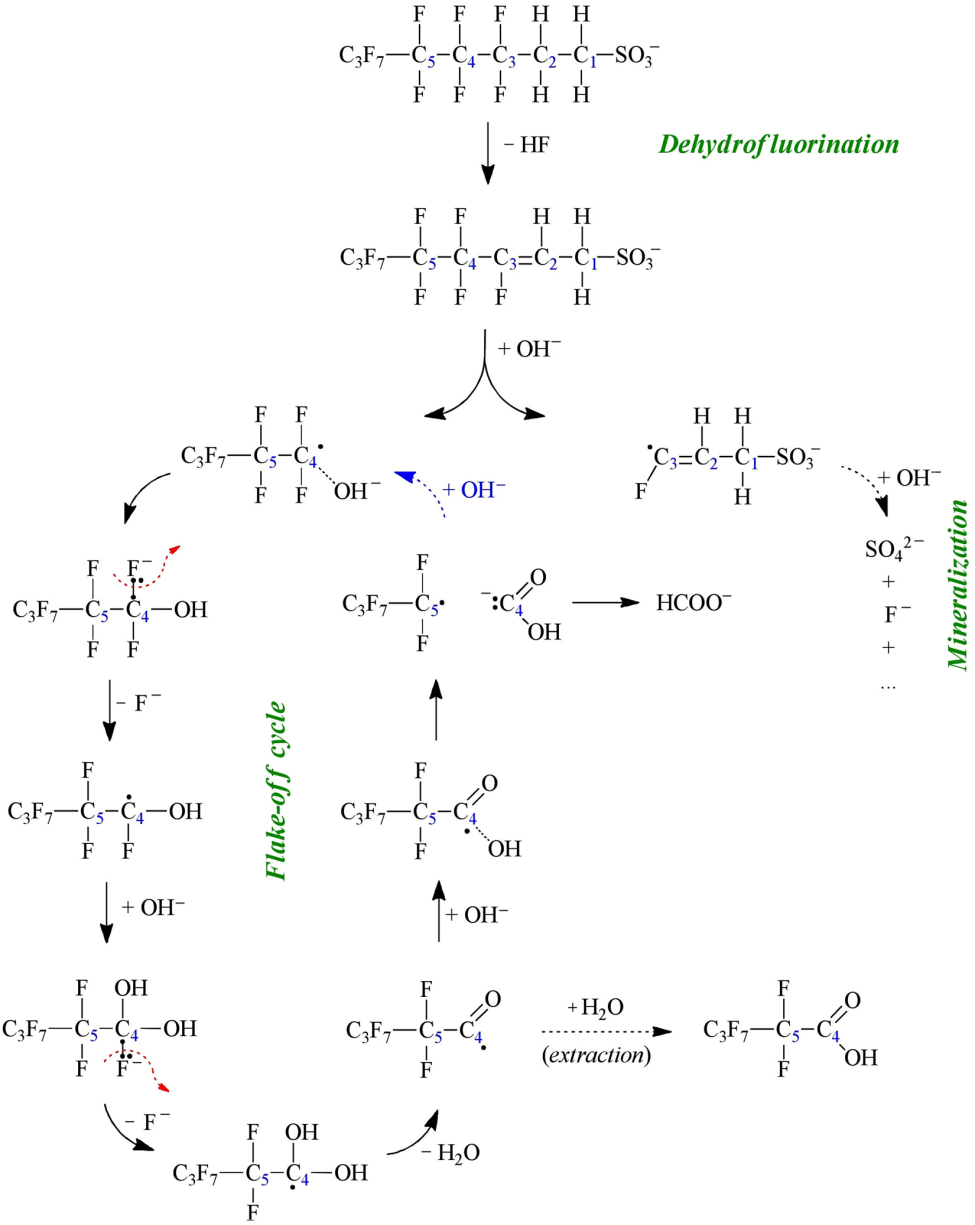
Proposed reaction mechanism for the MC destruction of 6:2FTS.

A possible mechanism for the reaction between 6:2FTS and KOH is proposed in Fig. 6. The first step is the 6:2FTS dehydrofluorination through HF expulsion and consequent formation of a double bond between C_2_ and C_3_. In general, the MC destruction of perfluorosulfonates starts with sulfonate group detachment, independently from the employed co-milling reagent^35–37^. For such chemicals, it is hypothesized that the bond between perfluoroalkyl chain and polar group is the weakest one. The case of 6:2FTS is dissimilar: the presence of hydrogens in the chain conceivably introduces an additional fragile point in the structure. Moreover, hydrogenated C_1_ is less electrophilic than its homologue in perfluorinated surfactants, so it is less receptive toward nucheophilic reagents (e.g. OH^−^) and polar group detachment is less probable. Direct evidence for dehydrofluorination could not be found in the present study. But, this reaction step is compatible with the intermediates detected by LC-MS-MS. More importantly, Nomura *et al*.^57^ already proved that γ-hexachlorocyclohexane is degraded by high energy ball milling with CaO through a dehydrochlorination pathway that leads to formation of polychlorobenzene intermediates, which are further destroyed by the well-studied MC dechlorination and carbonization mechanism. Here it is supposed that the dehydrofluorination is the main degradation process that causes 6:2FTS transformation into a 2-octenesulfonate.

The second step is the fragmentation of the 2-ene-compound by homolytic rupture of C_3_‒C_4_ bond, probably induced by OH^−^. Since PFPeA is the observed intermediate with the longest chain, it is inferable that the C_3_‒C_4_ bond is quantitatively broken up during high energy ball milling. Several studies demonstrate that organic radicals are commonly formed as consequence of MC treatment, also for perfluorinated compounds^58,59^. Formation of radical fragments is corroborated by the rapid build-up of fluorides, which follows the 6:2FTS degradation kinetics. Such fast reaction rate can be explained only by generation of very reactive species, i.e. radicals. Being a nucheophile, OH^−^ attacks the most positively charged and sterically available carbon of the 2-octenesulfonate carbon chain, i.e. C_4_ bounded with two fluorine atoms. This fact, together with the mechanical action, determines bond breakage.

The attacking hydroxide then remains bound to the perfluorinated moiety by C_4_‒OH bond formation. Its negative charge is taken by fluorine, which detaches as fluoride. This OH^−^ attack followed by F^−^ expulsion is repeated for the second fluorine to generate an unstable radical intermediate with two hydroxyl groups attached to the same carbon (C_4_). The subsequent reaction is very likely the dehydration of such radical to form a ketonic group. This reaction has two advantages: it expels one oxygen from the radical, and the presence of C=O stabilizes it by resonance. The observed perfluorocarboxylate intermediates (Fig. 5) are probably quantitatively generated by hydration of the ketyl radical during the water extraction procedure. Finally, the radical is attacked by another hydroxide, and the excessive negative charge induces C_4_ moiety detachment to give origin to a shorter perfluorinated radical and a carbanion, which transforms into formate by hydrogen rearrangement. The perfluoroalkyl chain, with one less CF_2_ moiety, undergoes to the same “flake-off” cycle until total mineralization.

The hydrogenated fragment with sulfonate group (C_3_H_3_FSO_3_^−^) generated by rupture the C_3_‒C_4_ bond of the 2-ene-compound undergoes incomplete destruction, as demonstrated by partial sulphate recovery. However, the mechanism is probably different from the flake-off cycle due to the presence of hydrogen, which reasonably cannot capture electrons like fluorine atoms. Consequently, diversely from sulphates (which were detected by IC) and fluorides (as highlighted by the kinetic analysis in paragraph 3.3), it is only hypnotizable that the mineralization might generate formate as end product. Ascertain C_3_H_3_FSO_3_^−^ degradation mechanism is beyond the scope of the present study, which is aimed to assess the effective and safe MC mineralization of fluorotelomers.

The proposed mechanism implies that the most noxious moiety of 6:2FTS (i.e. the perfluorinated chain) is mineralized in an effective manner by the flake-off cycle. The possibility of toxic by-products release is limited by the fact that all organic intermediates have a radical nature, so they quickly react with KOH (which is massively present in the reaction mixture).

### Kinetic analysis

Direct identification of intermediate compounds in MC reaction is a difficult task. As mentioned in the previous paragraph, they are radical species with short half-lives and are easily consumed in air (in particular, by oxygen). A useful approach to study MC destruction reaction is to determine reagent and product kinetics in ~100% conversion reactions (in stoichiometric conditions) to infer possible intermediate concentration trends by mechanism modelling^37^. Yet, the case of 6:2FTS is almost unique: fluorides exhibit a peculiar trend, which indicates that their accumulation might be caused by reactions that occurs in various moments (Fig. 2). Precisely, their amount (respect to their stoichiometric quantity) shows a rapid development in the first 20 min of the MC reaction (along with 6:2FTS destruction), but reveals a very slow increase during the final part (20–60 min). This peculiar trend called for a deeper understanding of the reaction mechanism. To this aim, a kinetic analysis based on a simplified reaction model was carried out. Specifically, the above-described mechanism is condensed into the following chemical reactions:

Dehydrofluorination-fragmentation (DHF)

1 $$6:\mathrm{2FTS}\mathop{\to }\limits^{\,{k}_{DHF}\,}{{\rm{C}}}_{5}{{\rm{F}}}_{{\rm{11}}}{\rm{OH}}+{{\rm{C}}}_{3}{{\rm{H}}}_{3}{{\rm{FSO}}}_{3}^{-}+{{\rm{F}}}^{-}$$

Flake-off mineralization (FOM)

2 $${{\rm{C}}}_{5}{{\rm{F}}}_{{\rm{11}}}{\rm{OH}}\mathop{\to }\limits^{\,{k}_{FOM}\,}{{\rm{C}}}_{4}{{\rm{F}}}_{9}{\rm{OH}}+{{\rm{2F}}}^{-}+{{\rm{HCOO}}}^{-}$$

3 $${{\rm{C}}}_{2}{{\rm{F}}}_{5}\mathrm{OH}\,\mathop{\to }\limits^{\,{k}_{FOM}\,}{{\rm{CF}}}_{3}{\rm{OH}}+{{\rm{2F}}}^{-}+{{\rm{HCOO}}}^{-}$$

CF_3_ mineralization (CF_3_M)

4 $${{\rm{CF}}}_{3}{\rm{OH}}\mathop{\to }\limits^{\,{k}_{C{F}_{3}M}\,}3{{\rm{F}}}^{-}+{{\rm{HCOO}}}^{-}$$

Polar fragment (C_3_H_3_FSO_3_^−^) mineralization (PFM)

5 $${{\rm{C}}}_{3}{{\rm{H}}}_{3}{{\rm{FSO}}}_{3}^{-}\mathop{\to }\limits^{\,{k}_{PFM}\,}{{\rm{SO}}}_{4}^{-}+{{\rm{F}}}^{-}+\ldots $$

Since hydroxide amount is preponderant in the reaction system, its concentration can be considered constant during the reaction, therefore it is not included among key compounds of the model. Accordingly, mass and charge balances are not satisfied. Each reaction is presumed to be of the first order; this has been found to be a valid assumption for MC destruction of organic pollutants, included perfluorinated compounds^37,60^.

It is noteworthy that each degradation reaction of the model has fluorides among the products. In other words, each reaction contributes to fluoride build-up at a different rate. As a result, the kinetic analysis is useful to identify separately the contributions of each reaction step to fluoride accumulation, and in this way corresponding reaction rates can be estimated.

Figure 2 shows the components of fluoride production given by each reaction of the model. The first step is the dehydrofluorination (DHF, eq. 1). Its kinetics is univocally established by 6:2FTS depletion rate, so the corresponding fluoride contribution is accordingly determined. Mineralization by flake-off (FOM, eqs 2–3) causes the greatest molar production of fluorides, hence its fluoride generation has to reach the stoichiometric yield in rapid manner, otherwise it would be impossible to fit the experimental curve. In particular, the best fit highlights that FOM fluorides are mainly produced in the early stage of the MC reaction (<10 min). Since flake-off is reasonably a sequential process, CF_3_ mineralization (CF_3_M, eq. 4) rate is supposed to be equal to or slower than that of FOM. Besides, because it releases 3 F^−^ per each 6:2FTS, such step also has to arrive quickly to its maximum yield to achieve a satisfactory fitting of experimental data. Calculation shows that it chiefly contributes to fluoride build-up in the middle stage of the reaction (10–20 min). All mentioned reaction steps (eqs 1–4) are rather fast, therefore the mineralization of the polar fragment (PFM, eq. 5) is the sole process that is likely responsible for the slow fluoride generation in the late phase of the MC treatment (>20 min).

Kinetic constants were estimated on the basis of the above-mentioned hypotheses (Table 1). Calculation results indicate that each flake-off cycle (i.e. mineralization of a CF_2_) is near 10 times faster than dehydrofluorination, thus confirming the high reactivity of radical intermediates. Surprisingly, CF_3_ defluorination rate is more than 10 times slower than that of CF_2_. A possible explanation is the reduced reactivity of CF_3_, when the majority of electron-withdrawing fluorine atoms have already flaked off. The very slow reactivity of the hydrogenated polar moiety by PFM reaction is even more unexpected, for two reasons: C‒H bond is less energetic than C‒F, and previous studies indicates that the C‒SO_3_^−^ bond is preferably broken in perfluorinated molecules under ball milling with KOH^35,36^. However, hydrogen is not an electron-withdrawing atom, so it cannot accept easily negative charges from OH^−^. For this reason, it possibly stabilizes the bond between the alkyl chain and the polar group. Moreover, PFM rate indicates that after 60 min milling ~50% fluorides are released from the polar fragments. The 10% sulphate recovery from reaction products is consistent with this figure, and substantiates the very slow destruction rate of the polar fragment.

**Table 1:** Kinetic constants of the 6:2FTS MC destruction model.

Reaction	Kinetic constant (min^−1^)
DHF (eq. 1)	0.28
FOM (eqs 2, 3)	2.00
CF_3_M (eq. 4)	0.17
PFM (eq. 5)	0.01

The flake-off reaction rate (included CF_3_ mineralization) is important to predict possible generation of organic fluorinated by-products during the MC treatment. The kinetic analysis clearly demonstrates that such process is very rapid and occurs along with 6:2FTS degradation. This corroborates that the MC destruction of 6:2FTS is a safe and economically viable process for C_6_ fluorotelomers disposal.

## Conclusions

Very persistent fluorotelomer surfactants are among the possible substitutes of long chain PFASs for the manufacture of fluorine-based products. Conceivably, their concentration in industrial waste will increase in the next years, demanding for adequate disposal. The present work proved that high energy ball milling with KOH is an efficacious method for mineralization of 6:2FTS, suggesting that such treatment can be applied to all fluorotelomers. Stoichiometric fluoride recovery (together with other analytical outcomes) corroborates the entire mineralization in less than 1 h milling. On the basis of experimental results, a 6:2FTS destruction mechanism was proposed. MC reaction begins with molecule cleavage in two fragments, i.e. the perfluorinated chain and the polar moiety with hydrogenated alkyl chain. The perfluorinated chain is dismantled by CF_2_ flake-off through a radical mechanism to generate fluorides and formates. A kinetic study based on the mechanism was also performed. This highlights that the flake-off is a very rapid process, implying that the chance of noxious fluorinated gaseous compounds formation is low, if ball milling is carried out for sufficiently long time. Such results indicate that MC destruction is a viable technology to destroy very persistent fluorotelomers in solid waste. In addition, they suggest, together with previous research on other PFAS MC destruction^35,36^, that co-milling with KOH can be likely used to mineralize any possible long chain substitute that will be employed in the future. Further research on MC treatment of fluorotelomer-containing industrial waste is programmed.

## Materials and Methods

### Materials

Potassium salt of 6:2FTS (C_6_F_13_C_2_H_4_SO_3_K, CAS No. 59587-38-1, 99% NMR purity) was provided by DuPont (USA), while potassium hydroxide (KOH, >90% purity) was purchased from J&K Chemical China Ltd (China). Ammonium acetate (NH_4_Ac, >99% purity, Sigma-Aldrich, USA) was employed to prepare LC-MS-MS mobile phase. Deionized water was prepared by a Milli-Q system (Millipore, USA), while the other utilized solvents were of HPLC grade (J.T. Baker Inc., USA).

### Milling experiment

6:2FTS (0.2 g) and KOH (4.6 g) were put in a 100 mL stainless steel jar with 180 g stainless steel balls (Ø5 mm), and co-milled by a planetary ball mill (QM-3SP2, Nanda Instrument and Equipment Factory, China). Main disk rotation speed was set to 275 rpm, with −2:1 jar rotation-main disk revolution ratio. During milling, the rotation direction reversed after every 30 min.

Milled samples (60 mg) underwent ultrasonic extraction for 30 min, using 50 mL deionized water; extracts were filtered with PTFE filters (0.22 μm). Fluoride and sulphate ions in products were quantified by ion chromatography (IC, ICS2000, Dionex) using an AG18 ion-exchange column with 0.025 M potassium hydroxide mobile phase at a flow rate of 1.0 mL min^−1^. Residual 6:2FTS and reaction intermediates were determined by LC (Ultimate 3000, Dionex Co., USA) with a reverse phase column (3.0 × 150 mm, 3.5 μm, XBridge^TM^, Waters, USA), followed by a MS-MS detector (API 3200, AB SCIEX, USA). Methanol and 0.05 M ammonium acetate aqueous solution were used for the mobile phase, with a flow rate of 0.3 mL min^−1^. Negative ionization and multiple reaction monitoring modes were applied to analyse the target compounds.

### Milled residue characterization

Crystalline phases in milled product were identified by X-ray diffraction (SmartLab, Rigaku, Japan), utilizing Cu Kα radiation in the 2θ range of 10°–80° at speed of 10° min^−1^. ^19^F nuclear magnetic resonance spectroscopy (JNM-ECA600, JEOL, USA) was performed using CD_3_OH as solvent to investigate fluorine mineralization. Chemical bond transformations were assessed by Fourier transform infrared spectroscopy (FTSA3000, Digilab, USA) with the KBr disk method, varying wavenumber from 400 to 4000 cm^−1^. X-ray photoelectron spectroscopy (PHI Quantera SXM, ULVAC-PHI, Japan) was employed to observe F and C electronic state changes.
